# Information Flow in Networks and the Law of Diminishing Marginal Returns: Evidence from Modeling and Human Electroencephalographic Recordings

**DOI:** 10.1371/journal.pone.0045026

**Published:** 2012-09-18

**Authors:** Daniele Marinazzo, Guorong Wu, Mario Pellicoro, Leonardo Angelini, Sebastiano Stramaglia

**Affiliations:** 1 Faculty of Psychology and Educational Sciences, Department of Data Analysis, Ghent University, Belgium; 2 Key Laboratory for NeuroInformation of Ministry of Education, School of Life Science and Technology, University of Electronic Science and Technology of China, Chengdu, China; 3 Dipartimento di Fisica, Università degli Studi di Bari and INFN Bari, Bari, Italy; University of Zaragoza, Spain

## Abstract

We analyze simple dynamical network models which describe the limited capacity of nodes to process the input information. For a proper range of their parameters, the information flow pattern in these models is characterized by exponential distribution of the incoming information and a fat-tailed distribution of the outgoing information, as a signature of the law of diminishing marginal returns. We apply this analysis to effective connectivity networks from human EEG signals, obtained by Granger Causality, which has recently been given an interpretation in the framework of information theory. From the distributions of the incoming versus the outgoing values of the information flow it is evident that the incoming information is exponentially distributed whilst the outgoing information shows a fat tail. This suggests that overall brain effective connectivity networks may also be considered in the light of the law of diminishing marginal returns. Interestingly, this pattern is reproduced locally but with a clear modulation: a topographic analysis has also been made considering the distribution of incoming and outgoing values at each electrode, suggesting a functional role for this phenomenon.

## Introduction

Most social, biological, and technological systems can be modeled as complex networks, and display substantial non-trivial topological features [1], [2]. Moreover, time series of simultaneously recorded variables are available in many fields of science; the inference of the underlying network structure, from these time series, is an important problem that received great attention in the last years. A method based on chaotic synchronization has been proposed in [3], a method based on model identification has been described in [4]. Use of a phase slope index to detect directionalities of interactions has been proposed in [5].

The inference of dynamical networks is also related to the estimation, from data, of the flow of information between variables, as measured by the transfer entropy [6], [7]. Wiener [8] and Granger [9] formalized the notion that, if the prediction of one time series could be improved by incorporating the knowledge of past values of a second one, then the latter is said to have a *causal* influence on the former. Initially developed for econometric applications, Granger causality has gained popularity also among physicists (see, e.g., [10]–[15]) and eventually became one of the methods of choice to study brain connectivity in neuroscience [16]. Multivariate Granger causality may be used to infer the structure of dynamical networks from data as described in [17]. It has been recently shown that for Gaussian variables Granger causality and transfer entropy are equivalent [18], and this framework has also been generalized to other probability densities [19]. Hence a weighted network obtained by Granger causality analysis can be given an interpretation in terms of flow of information between different components of a system. This way to look at information flow is particularly relevant for neuroscience, where it is crucial to shed light on the communication among neuronal populations, which is the mechanism underlying the information processing in the brain [20]. Furthermore, recent studies have investigated the economics implications of several network types mapping brain function [21], [22].

In many situations it can be expected that each node of the network may handle a limited amount of information. This structural constraint suggests that information flow networks should exhibit some topological evidences of the law of diminishing marginal returns [23], a fundamental principle of economics which states that when the amount of a variable resource is increased, while other resources are kept fixed, the resulting change in the output will eventually diminish [24], [25]. The purpose of this work is to introduce a simple dynamical network model where the topology of connections, assumed to be undirected, gives rise to a peculiar pattern of the information flow between nodes: a fat tailed distribution of the outgoing information flows while the average incoming information flow does not depend on the connectivity of the node. In the proposed model the units, at the nodes the network, are characterized by a transfer function that allows them to process just a limited amount of the incoming information. We show that a similar behavior is observed in another network model, which describes in a different fashion the law of diminishing marginal returns. Moreover, we also propose an exactly solvable Ising model on sparse networks, in the limit of an infinite number of nodes, whose behavior may be seen in the light of the law of diminishing marginal returns. Finally we show that this relevant topological feature is found as well in real neural data.

## Materials and Methods

We implement three models on different network structures. Then we analyze human EEG data.

### Model 1

The first model we propose is as follows. Given an undirected network of 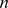 nodes and symmetric connectivity matrix 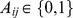, to each node we associate a real variable 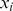 whose evolution, at discrete times, is given by:
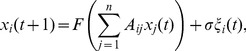
(1)where 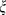 are unit variance Gaussian noise terms, whose strength is controlled by 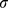; 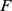 is a transfer function chosen as follows:
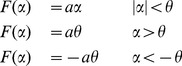
(2)where 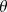 is a threshold value. This transfer function is chosen to mimic the fact that each unit is capable to handle a limited amount of information. For large 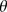 our model becomes a linear map. At intermediate values of 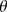, the nonlinearity connected to the threshold will affect mainly the mostly connected nodes (hubs): the input 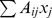 to nodes with low connectivity will remain typically sub-threshold in this case. We consider hierarchical networks generated by preferential attachment mechanism [26]. From numerical simulations of eqs. (1), we evaluate the linear causality pattern for this system as the threshold is varied. We verify that, in spite of the threshold, variables are nearly Gaussian so that we may identify the causality with the information flow between variables [18].

### Model 2

We also analyze the following model: to each node of an undirected network we associate the variable 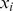 whose evolution is 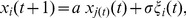
(3)where 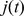 is a node chosen randomly, at each time 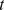, in the set of the neighboring nodes of 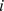. Equations (3) implement, in a different way from (1), the occurrence that nodes may handle a limited incoming information: at each time each node is influenced just by one other node.

### Model 3

As another example we consider a diluted Ising model on a directed network [27], [28], constructed as follows. The model is made of 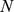 Ising spins 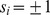, each connected (with coupling 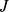) to 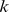 input spins, chosen at random among the 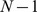 remaining spins. The number of incoming links for each spin, the in-degree 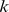, is independently sampled with probability 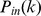, 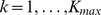, 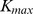 being the maximum value that 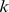 may assume. The dynamics of the system corresponds to parallel updating of Ising variables 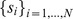:
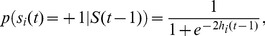
(4)where the local fields are given by
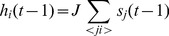
(5)where the sum is over the input spins of 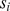, and 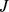 is the positive coupling. We will consider the limit 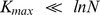: it is well known that input spins may be treated as independent stochastic variables in this limit: this makes simple the numerical evaluation of 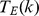, the transfer entropy from one input spin to a target spin of connectivity 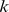 (see, e.g., [29]). For 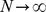 the out-degree of spins, 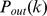, is a Poisson distribution with parameter



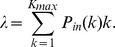



The input flow of information for a spin with in-degree 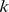 is
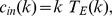
whilst the average information flow outgoing a spin of out-degree 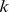 is given by



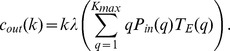



The distribution of 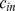 in the whole system is.
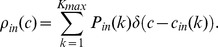



### Human EEG Data

As a real example we consider electroencephalogram (EEG) data. We used recording obtained at rest from 10 healthy subjects. During the experiment, which lasted for 15 min, the subjects were instructed to relax and keep their eyes closed. To avoid drowsiness, every minute the subjects were asked to open their eyes for 5 s. EEG was measured with a standard 10–20 system consisting of 19 channels [5]. Data were analyzed using the linked mastoids reference, and are available from [30].

## Results

### Model 1

Concerning the first model, we compute the incoming and outgoing information flow from and to each node, 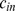 and 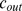, summing respectively all the sources for a given target and all the targets for a given source. Then we evaluate the standard deviation of the distributions of 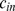 and 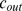, varying the realization of the preferential attachment network and running eqs. (1) for 10000 time points.

**Figure 1:**
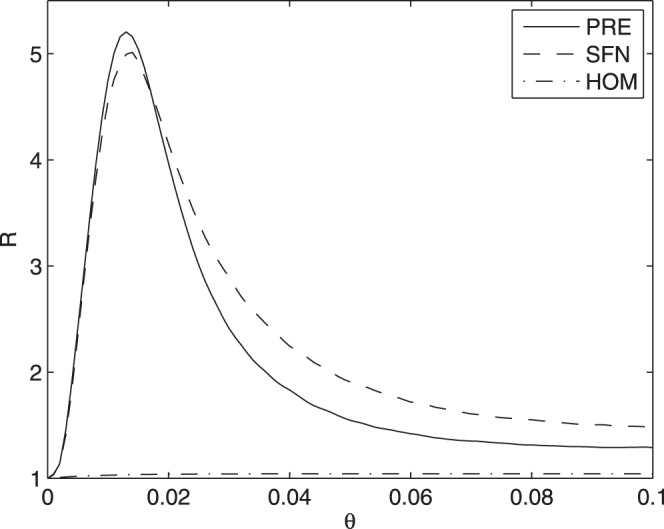
Modulation of R for different network architectures. The ratio between the standard deviation of 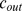 and those of 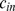, 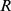, is plotted versus 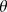 for the three architectures of network: preferential attachment (PRE), deterministic scale free (SFN) and homogeneous (HOM). The parameters of the dynamical system are 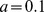 and 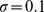. Networks built by preferential attachment are made of 30 nodes and 30 undirected links, while the deterministic scale free network of 27 nodes is considered. The homogeneous networks have 27 nodes, each connected to two other randomly chosen nodes.

In figure 1 we depict 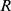, the ratio between the standard deviation of 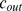 over those of 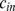, as a function of the 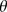. As the threshold is varied, we encounter a range of for which the distribution of 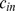 is much narrower than that of 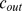. In the same figure we also depict the corresponding curve for deterministic scale free networks [31], which exhibits a similar peak, and for homogeneous random graphs (or Erdos-Renyi networks [32]), with 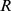 always very close to one. The discrepancy between the distributions of the incoming and outgoing causalities arises thus in hierarchical networks. We remark that, in order to quantify the difference between the distributions of 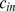 and 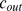, here we use the ratio of standard deviations but qualitatively similar results would have been shown using other measures of discrepancy.

**Figure 2:**
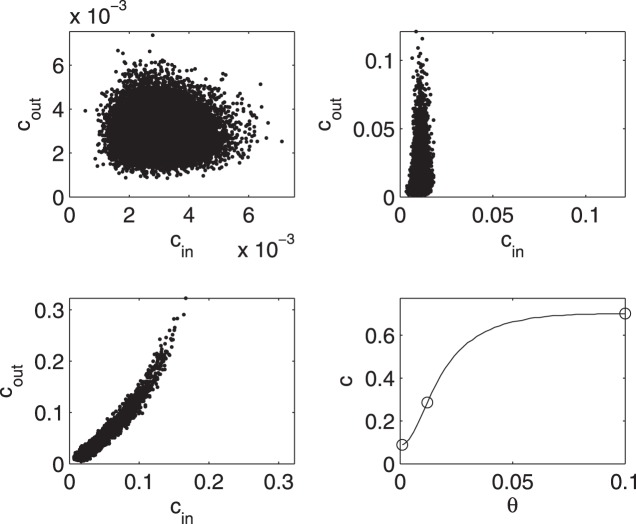
Incoming and outgoing information and coupling. Scatter plot in the plane 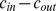 for undirected networks of 30 nodes and 30 links built by means of the preferential attachment mechanism. The parameters of the dynamical system are 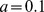 and 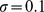. The points represent the nodes of 100 realizations of preferential attachment networks, each with 10 simulations of eqs. (1) for 10000 time points. (Top-left) Scatter plot of the distribution for all nodes at 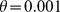. (Top-right) Contour plot of the distribution for all nodes at 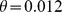. (Bottom-left) Scatter plot of the distribution for all nodes at 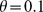. (Bottom-right) The total causality (obtained summing over all pairs of nodes) is plotted versus 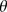; circles point to the values of 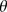 in the previous subfigures.

**Figure 3:**
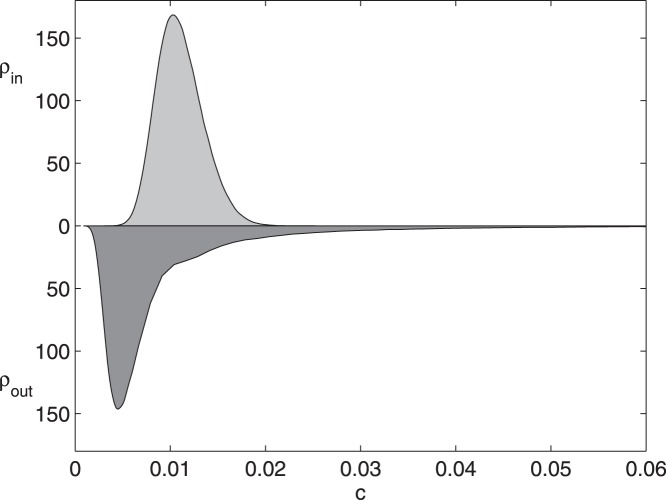
Distributions of information flow for the preferential attachment network. For the preferential attachment network, at 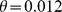, the distributions (by smoothing spline estimation) of 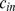 and 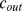 are depicted. Units on the vertical axis are arbitrary.

In figure 2 we report the scatter plot in the plane 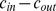 for preferential attachment networks and for some values of the threshold. The distributions of 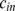 and 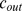, with 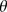 equal to 0.012 and corresponding to the peak of figure 1, are depicted in figure 3: 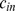 appears to be exponentially distributed around a typical value, whilst 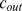 shows a fat tail. In other words, the power law connectivity, of the underlying network, influences just the distribution of outgoing causalities.

**Figure 4:**
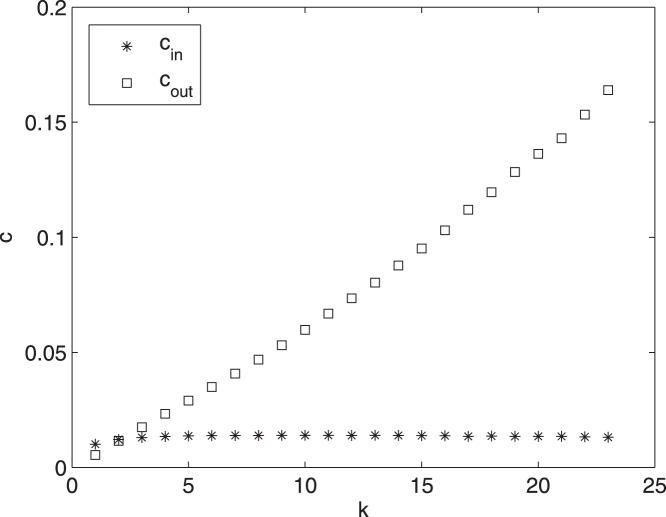
Information flow versus connectivity. In the ensemble of preferential attachment networks of figure (2), at 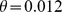, 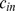 and 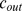 are averaged over nodes with the same connectivity and plotted versus the connectivity.

In figure 4 we show the average value of 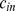 and 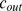 versus the connectivity 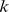 of the network node: 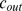 grows uniformly with 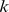, thus confirming that its fat tail is a consequence of the power law of the connectivity. On the contrary 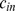 appears to be almost constant: on average the nodes receive the same amount of information, irrespective of 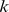, whilst the outgoing information from each node depends on the number of neighbors.

It is worth mentioning that since a precise estimation of the information flow is computationally expensive, our simulations are restricted to rather small networks; in particular the distribution of 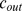 appears to have a fat tail but, due to our limited data, we can not claim that it corresponds to a simple power-law.

### Model 2

**Figure 5:**
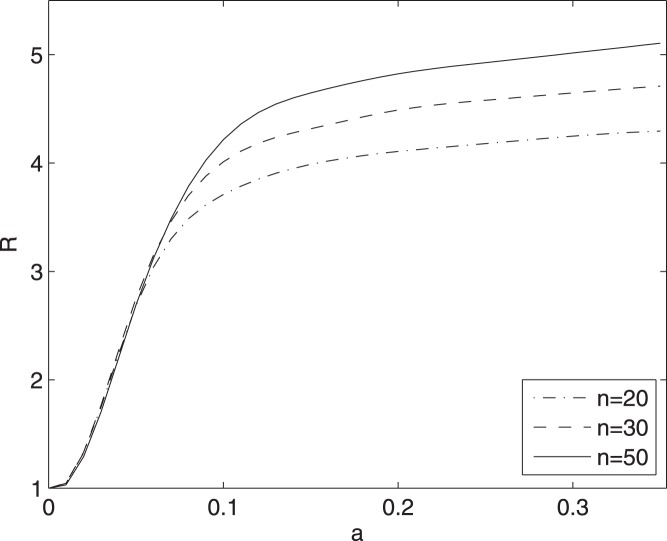
Influence of the network size on R. For the model (3) the ratio 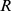, between the standard deviation of 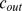 and those of 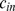, is depicted versus 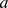. Preferential attachment networks, of 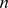 nodes and 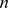 links, are considered.

A fat tail in the distribution of 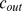 is observed also in model 2: in figure 5 we depict 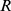 as a function of 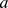, for preferential attachment networks and for different size of the networks: the discrepancy between the distributions of 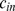 and 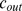 increases as the size of the network grows while keeping 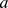 fixed.

### Model 3

**Figure 6:**
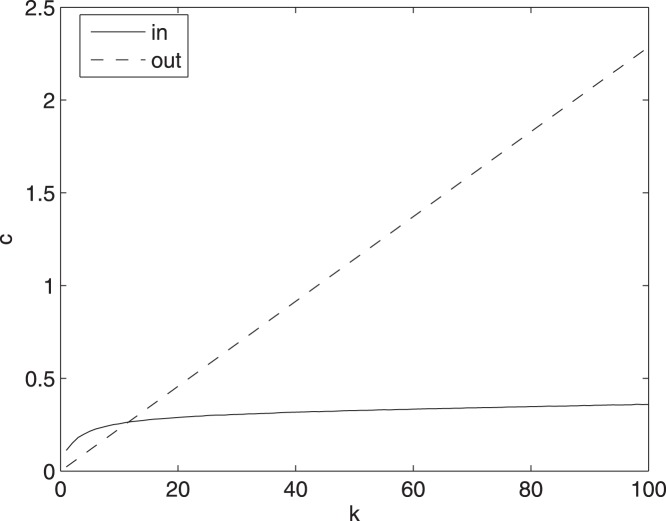
Information flow distributions for the Ising model. The total transfer entropy versus the in-degree and the out-degree for the Ising model.

As already stated, model 3 is exactly solvable in the limit 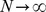. In figure 6 we depict 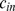 and 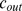 versus 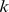, for a power law distribution for connectivity 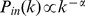, 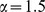, 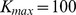 and 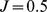. The incoming information flow tends to saturate for spins with large in-degree.

**Figure 7:**
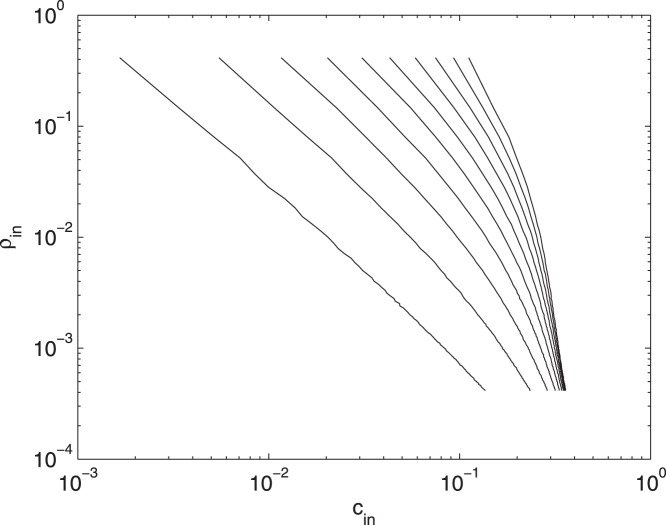
Modulation of incoming information for the Ising model. The distribution of 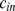, for the Ising model, with 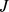 varying from 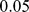 to 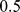 with step 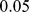 (from the left to the right).

In figure 7 we depict 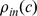 for several values of 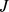 corresponding to a power law distribution for in-degree of spins characterized by 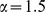. For low 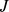 the distribution of 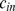 appears to be a power law as the in-degree distribution: 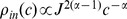 at small 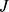. Increasing 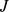, the distribution tends to became exponential, in spite of the power law of input connectivity. These results are robust w.r.t. changes in the exponent 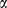.

### EEG Data

For each subject we considered several epochs of 4 seconds in which the subjects kept their eyes closed. For each epoch we computed multivariate Kernel Granger Causality [15] using a linear kernel and a model order of 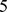, determined by leave-one-out cross-validation. We then pooled all the values for information flow towards and from any electrode and analyzed their distribution.

**Figure 8:**
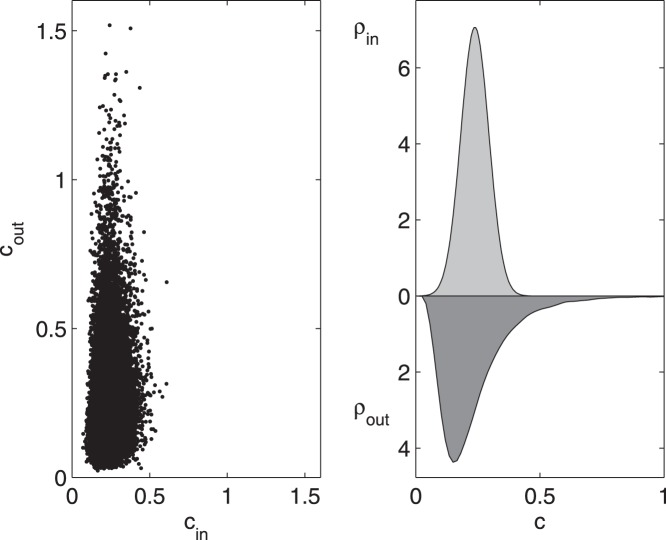
Incoming and outgoing information for EEG data. For the EEG data the distributions of 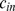 and 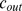 are depicted in a scatter plot (left) and in terms of their distributions, obtained by smoothing spline estimation (right).

In figure 8 we plot the incoming versus the outgoing values of the information flow, as well as the distributions of the two quantities: the incoming information seems exponentially distributed whilst the outgoing information shows a fat tail. These results suggest that overall brain effective connectivity networks may also be considered in the light of the law of diminishing marginal returns.

**Figure 9:**
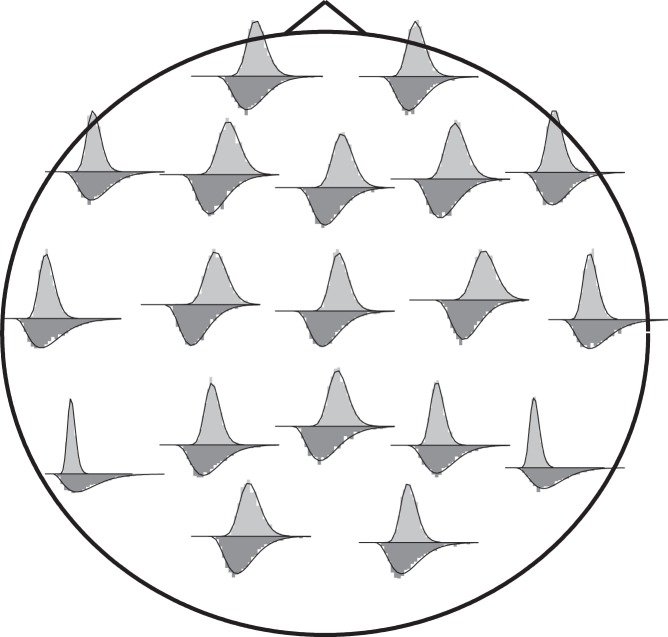
Topological probability distributions. The distributions for incoming (above, light grey) and outgoing (below, dark grey) information at each EEG electrode displayed on the scalp map (original binning and smoothing spline estimation).

**Figure 10:**
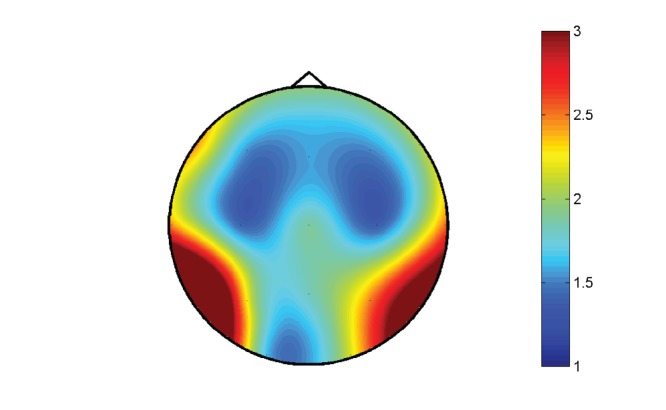
Topographic map of R. The distribution on the scalp of 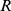, the ratio between the standard deviations of the distributions of outgoing and incoming information, for EEG data.

More interestingly, this pattern is reproduced locally but with a clear modulation: a topographic analysis has also been made considering the distribution of incoming and outgoing causalities at each electrode. In figure 9 we show the distributions of incoming and outgoing connections corresponding to the electrodes locations on the scalp, and in figure 10 the corresponding map of the parameter 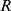; the law of diminishing marginal returns seems to affect mostly the temporal regions. This well defined pattern suggests a functional role for the distributions. It is worth to note that this pattern has been reproduced in other EEG data at rest from 9 healthy subjects collected for another study with a different equipment.

## Discussion

In this work we have pointed out that the pattern of information flow among variables of a complex system is the result of the interplay between the topology of the underlying network and the capacity of nodes to handle the incoming information. Implementing two simple toy models on different network structures, we have shown that they may exhibit the law of diminishing marginal returns for a suitable choice of parameters: the presence of nodes with different in-degree is a fundamental ingredient for these phenomena. Our simulations for these two models are restricted to rather small networks, due to the computational burden. However to address this issue we have also proposed an Ising model on a sparse network, which can be exactly solved in the limit of an infinite number of nodes; a similar behavior emerged as well in this case.

The analysis of a real EEG data-set has shown that similar patterns exist for brain signals and could have a specific functional role. We remark that the distribution of in-degree in resting state fMRI directed networks has been observed to fit an exponentially truncated power law [33]; in the same study the architecture of directed networks was presented as a complement to the same work performed in anatomical and functional connectivity.

Apart from fMRI, there is an increasing interest in investigating resting state networks from EEG recordings [34]. The findings of our study could then represent an additional feature to consider in these networks.

The study of information flow mechanisms is crucial in brain research, and effective methods to mine the information flow pattern from data have been recently introduced. Recently interesting contributions, towards a better understanding of communications in brain, have been provided [35]. Our results, thus, may be relevant to get a better characterization of the topology of brain networks.

In general, evidences of the law of diminishing marginal returns are related to the presence of units which are close to be receiving the maximal amount of information that they can process. A similar interpretation may apply in neuroscience. Indeed the brain is an expensive part of human body, and the organization of brain networks can be explained by a parsimonious drive; it has been proposed that connectomes organization corresponds to a trade-off between minimizing costs and the emergence of functional connectivity between multiple neural populations [22]. This economical principle in brain networks may also be connected to the presence, under particular circumstances, of brain units receiving the maximal amount of information in input. Such situations will display evidences of the law of diminishing marginal returns and should be put in evidence by the proposed analysis.

We should as well mention that there are other measures of directed brain connectivity, such as Directed Transfer Function, Partial Directed Coherence and Phase Slope Index, for which the interpretation in terms of information flow is still debated [36]. On the other hand we verified that a significant discrepancy between the distributions of incoming and outgoing connectivities holds also for these methods. Furthermore, bivariate measures do not display this asymmetry of the distributions of 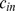 and 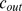: this is not surprising, indeed it is well known that bivariate causality also account for indirect interactions, see e.g. [17]. Here we limited ourselves to linear information flow; the amount of nonlinear information transmission and its functional roles are not clear [37]. It will be interesting to investigate these phenomena also in the nonlinear case.
